# Single Channel Image Enhancement (SCIE) of White Blood Cells Based on Virtual Hexagonal Filter (VHF) Designed over Square Trellis

**DOI:** 10.3390/jpm12081232

**Published:** 2022-07-28

**Authors:** Shahid Rasheed, Mudassar Raza, Muhammad Sharif, Seifedine Kadry, Abdullah Alharbi

**Affiliations:** 1 Department of Computer Science, COMSATS University Islamabad, Wah Campus, Islamabad 47040, Pakistan; shahidfordew@gmail.com (S.R.); muhammadsharifmalik@yahoo.com (M.S.); 2 Department of Applied Data Science, Noroff University College, 4612 Kristiansand, Norway; skadry@gmail.com; 3 Department of Information Technology, College of Computers and Information Technology, Taif University, Taif 21944, Saudi Arabia; amharbi@tu.edu.sa

**Keywords:** WBC, hexagonal filters, virtual pixels, image enhancement, grayscale images

## Abstract

White blood cells (WBCs) are the important constituent of a blood cell. These blood cells are responsible for defending the body against infections. Abnormalities identified in WBC smears lead to the diagnosis of disease types such as leukocytosis, hepatitis, and immune system disorders. Digital image analysis for infection detection at an early stage can help fast and precise diagnosis, as compared to manual inspection. Sometimes, acquired blood cell smear images from an L2-type microscope are of very low quality. The manual handling, haziness, and dark areas of the image become problematic for an efficient and accurate diagnosis. Therefore, WBC image enhancement needs attention for an effective diagnosis of the disease. This paper proposed a novel virtual hexagonal trellis (VHT)-based image filtering method for WBC image enhancement and contrast adjustment. In this method, a filter named the virtual hexagonal filter (VHF), of size 3 × 3, and based on a hexagonal structure, is formulated by using the concept of the interpolation of real and square grid pixels. This filter is convolved with WBC ALL-IBD images for enhancement and contrast adjustment. The proposed filter improves the results both visually and statically. A comparison with existing image enhancement approaches proves the validity of the proposed work.

## 1. Introduction

WBC smear images [1] are examined by using peripheral blood samples to help physicians in the efficient diagnosis of disease. WBCs are the underpinning element of any immune system. In the human immune system [2], WBCs defend the body against invaders/viruses and infections. Infection present inside the WBCs can cause various abnormalities at different stages of the disease. Diseases such as hepatitis [3] immune system disorder (ISD) [4] and many other diseases are usually diagnosed from WBC smear analysis. Visual inspection of WBCs during image analysis is performed to identify infection or an infected region inside the nucleus. Infectious diseases cause an incline in white blood cell count inside the body due to the excretion of chemokines by the infection-causing bacteria. These chemokines and other bacterial products attract the WBC at the site of infection along with an increase in production from the stem cells.

These WBCs fight the infection in different biological ways.

**Figure 1:**
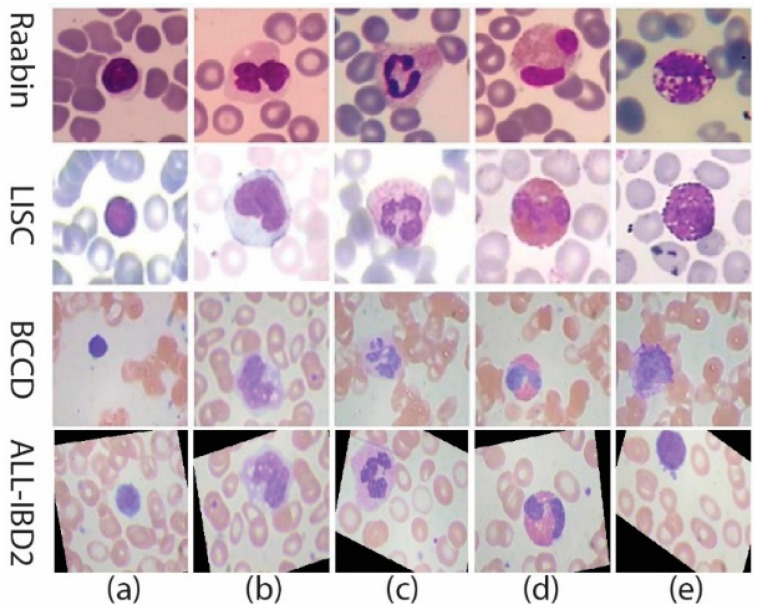
WBC classes: (**a**) eosinophil (**b**) neutrophil (**c**) basophil (**d**) monocyte (**e**) lymphocytes.

In the adaptive immune system, a major type of lymphocyte, named B-lymphocytes, induce “Y” shape terminal antibodies. When evaders (bacteria/virus) enter into the cell, these antibodies act to neutralize by completely coating the outer surface with other different types of antibodies. This phenomenon is called opsonization. Coated cell acts as a signal transmitter to other immune system cells, i.e., neutrophils. Neutrophils come into contact with the coated cell and digest the pathogen. As a result, infection (pathogen) enters into the cell body [5]. If the antibody structure is not healthy enough to destroy/digest the pathogen completely, it infects the inner cell body and the nucleus as well. Although the medical literature is extensive, here, we are only discuss the intensity-based visualization of WBCs. Infection generation depends upon the structure of the WBC nucleus. This infection may cause diseases of different types depending upon the infection class and level of infection. WBCs are mainly classified into two main classes: (a) Granulocytes and (b) Agranulocytes. In the Granulocytes the subtypes are (i) neutrophils, (ii) eosinophils, (iii) basophils, and (iv) lymphocytes. On the other hand, Agranulocytes consist of only one main type, that is Monocytes. Existing work contains various white blood datasets such as Raabin, LISC, BCCD, and ALL-IBD, with sub-classes [1] such as (a) Lymphocytes, (b) Monocyte, (c) Neutrophil (circular shaped nucleus) (d) Eosinophils, and (e) Basophil (as shown in Figure 1). Basophil is only 1% of the total WBC content in the body and has no contribution to infection generation. In this work, only four of the above types, excluding basophil, are used for WBC image analysis.

Preprocessing [6] is considered as a very essential part of WBC image analysis. Preprocessing includes the cropping of images to avoid the unwanted area from the background and the dehazing method. The subject image should be in a standard format to visualize the effect of the proposed method. During the preprocessing phase, unwanted regions and noisy parts are removed and the image is resized up to an acceptable size. In the medical domain, poor input data may lead to adverse predictions. Therefore, the input image is required to be preprocessed and normalized.

The WBC smear image enhancement makes it possible to visualize a cell’s inner structure more accurately and can help to identify the infectious region inside or outside the nucleus of a WBC. This improves the image’s contrast and brightness characteristics [7], reduces noise in the image, and sharpens the details. Some preprocessing techniques include image negation, histogram plotting, image subtraction and filtering [8], and interpolation [9]. The wiener filter [10] is a linear structure filter for image denoising. The wiener filter based on MSE-optimal stationary is used for the removal of noise and blueness from the image.

For a detailed assessment of WBC smear images, to indemnify the infectious part from the WBC images, a hexagonal structure-based method is proposed. The proposed virtual hexagonal trellis (VHT)-based filter enhances the images by reducing the blurring effect from the image and reduces the computational cost and information loss. The proposed approach enhances the images up to maximum visual clarity level. The virtual hexagonal base approach imparts a fruitful contribution to image enhancement as compared to classical methods. The presented VHT approach leads the image processing literature towards a new pathway for efficient image analysis. The proposed approach is quantified by comparing it with the square grid and is used to model VHT-based averaging filters. The square grid is a convention structure by which we acquire images from any image acquisition device. The proposed method is designed over the same square grid image with the conceptual implementation of hexagonal image processing without shifting the real pixels. The effective implementation of the VHT approach contributes to WBC image enhancement. The major contributions are mentioned as follows:

- The image dehazing process is used before the implementation of the VHT of the kernel for improving image enhancement quality. The dehazed image is then converted to a grayscale image;
- A convolution hexagonal structure base filter is proposed for image enhancement using a grayscale image. The proposed kernel structure is based on a virtual hexagonal trellis design over the square trellis;
- The comparison of previously presented approaches proves the robustness of the proposed model.

This manuscript is organized as follows: The introduction in Section 1 presents the basic concept of WBCs and hexagonal trellis. Related Work in Section 2 includes updated literature related to the presented technique. The proposed model is discussed in Section 3. Results and discussion related to the proposed work are discussed in Section 4. Finally, the conclusion is written with future direction in the last section. 

## 2. Related Work

The related work is divided into two portions—one covers existing image enhancement approaches, while second depicts an overview of the hexagonal image processing. Researchers have developed different methods for infection detection from the WBCs. Image enhancement is the key process in preprocessing automated bio-medical imaging [11,12], which leads scientists and doctors towards a better diagnosis. A weak edge enhancement operator (WEEO) [13] is introduced for the WBC fuzzy boundary. Hue saturation value is used by [14] for image enhancement. A new image enhancement technique named hybrid particle swarm optimization (PSO)-based contrast stretching (HPSO-CS) [15] is used, and requires more time compared to other deep learning methods [16]. The PSO is used to optimize the fitness function to rally the contrast and further details in the microscopic images by familiarizing the parameters as an influence on the image enhancement techniques. In this approach, to ease the segmentation process of acute blood leukemia, three contrast enhancement techniques are presented, i.e., partial contrast and bright and dark stretching [17] to improve the image quality. The discussed approach needs an auto segmentation mechanism for fast diagnosis. The triangle method based on the histogram equalization technique is used by [18,19] and constructs a straight line connecting the highest histogram value with the lowest histogram value. The highest and lowest histogram values turn the area of interest blurry and dark. Therefore, to overcome this problem, the distance between the manifest line and the histogram intensity values between (minimum histogram value and maximum histogram value) is then calculated. The interpolation or enhancement concept is introduced by [20] using Hex-Gabor processing for medic image processing, providing error-free enhanced images. This method is costly in terms of computational time and loss of information due to quantization during transformation. Radiological image features analysis using multi-resolution in [21] is presented for contrast enchantment, which pinned over the non-separable and multi-scale method. In this method, the local contrast enchantment method is improvised for medical images’ region enhancement. A high dynamic algorithm is presented for the conversion of the image from scene [22] (rectangular structure) to real world scene. The work [20,23] based on filtering-based image enhancement is presented by Gabor kernel in the hexagonal domain in view of the orthogonal directions in different orientations (mostly three). The Gabor wavelet on the hexagonal-sampled structure presented the optimal approximation of theoretical performance for enhanced quality image in [24], with a low computational cost of processing the images over the hexagonal grid. A hexagonal interpolation method with a half-shifted position of alternative row pixels method is introduced. During pixel shifting, in [25], information loss is a big problem for ultrasonic image enhancement using interlaced sampling using pixels in a square grid.

A variable lattice-based hexagonal discrete Fourier transform is derived from a hexagonal structure-based simulated display [26]. In this approach, a Voronoi cell is introduced with tentative intensity value and displayed over hexagonal patterns. From the literature, although previously presented methods have generated good WBC enhancement results, they still have some dark issues. The traditional methods based on square lattice includes the time taken, and some information lost is observed during the sampling or quantization phase. These methods require extra space when transformed from one square to hexagon-based lattice and vice versa. Considering these issues, we presented a hexagonal trellis base approach for WBC smear image enhancement, which rectifies issues in the traditional methods. The proposed work not only enhanced the WBC smear images, but is also helpful in other methods where enhanced images will be used for diagnosis purposes.

## 3. Material and Method

**Figure 2:**
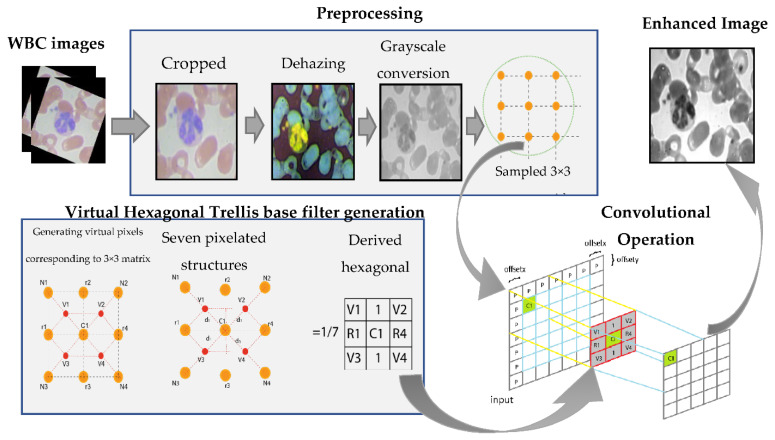
Block diagram of the proposed method and VHT-based filter for WBC image enhancement.

In this work, a virtual hexagonal trellis-based filter is derived and implemented for WBC image enhancement. Initially, the input images are preprocessed by applying a dehazing technique and an image compliment method. The preprocessed image is then convolved with the proposed VHT-based filter (as presented in Figure 2). The image of WBCs is often in blur form with dark contrast regions inside the cell body. The proposed VHT base filter uses the concept of the pixel interpolation method to refine the image.

The framework of the proposed methodology is depicted in Figure 2. It contains two steps: preprocessing and design and implementation of the VHT filter. In the first step, the input image is preprocessed (cropped, dehazed, and converted onto grayscale). In second step of proposed method, a VHT convolution filter is designed and convolved over WBC original images. After the convolution process, enhanced grayscale image is evaluated for quality assessment through performance evaluation parameters. The steps of the proposed work are discussed one by one in the accompanying subsections.

### 3.1. Preprocessing

In the preprocessing stage, image data are first normalized by avoiding extra parts from the background. This process starts with cropping and resizing of the image and is then trailed by dehazing and grayscale conversion of the WBC images.

#### 3.1.1. Image Cropping

**Figure 3:**
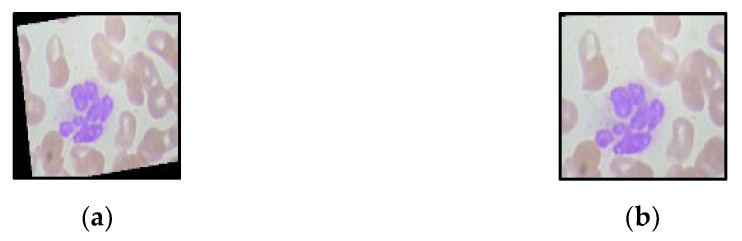
Image cropping (**a**) original image, (**b**) cropped and resized image.

Images collected from the L2-microscope contain a dark background. The images are cropped to evade dark backgrounds. The complete dataset is cropped and resized to maintain the normalization in data shown in Figure 3. For standard processing, the complete dataset is cropped and resized.

#### 3.1.2. Image Compliment

**Figure 4:**
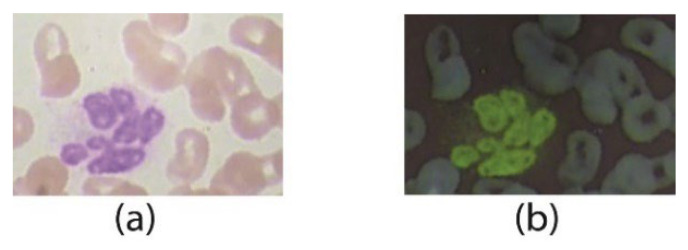
Image compliment (**a**) original image (**b**) complimented image.

While collecting WBC images, some noisy and unwanted dark regions are found in the cytoplasm. To remove the unwanted dark areas, the image complement method is applied. In this method, a single value of colored pixels in discrete form is presented in the true-color (RGB) image shown in Figure 4a, and an image is converted into its complimented image, as presented in Figure 4b. Without using the image compliment function, the cytoplasm region remains dark with other objects (i.e., platelets, antibodies, etc.) inside the cytoplasm, which affects the efficiency of the image enhancement process. The compliment process is mathematically written as:

(1) $$I^{c}(i,j)=\partial_{j\in \lambda (i,j)}\left(\partial_{cs\in (r,g,b)}\left(I_{s}^{c}\left(C\right)\right)\right)$$

where “λ” is the local patch in a color image, and $“I^{c}”$ is the complimented image. Where (*i*,*j*) are the coordinates of the image, “*C*” is the depth map, and “*∂*” is the local path and “s” represented the under exposure.

#### 3.1.3. Image Dehazing

**Figure 5:**
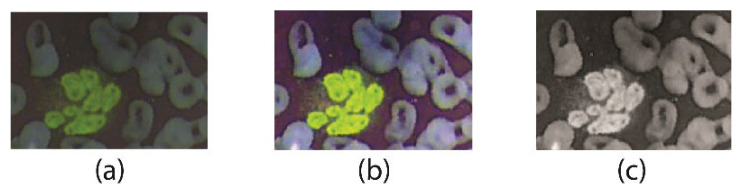
Image dehazing (**a**) image compliment (**b**) dehazed with inverse complimented image (**c**) grayscale image.

The dehazing technique is used for the removal of extra color intensities and unwanted dark shading which appear after the image compliment in true colors, as shown in Figure 5a. Using dehazing prior knowledge, hazy and dark parts from the cytoplasm of WBC images are removed to clear the nucleus and WBC’s cytoplasm region. At this stage, the WBC image is much more refined with the brightening of the dark areas in the cell region, as shown in Figure 5b. After this process, the entire focus is on WBCs and the inner part of the cell, i.e., the nucleus, for enhancement.

The dehazing scheme used in this work [27,28] deals with four pre-defined steps. In the first step, image depth map estimation is performed using the local path shown in Equation (1). In the second step, the over-covered atmospheric mask on the image is removed with maximum intensity values with local maxima presented in (2), where “I” is the image, “C” is the depth map function, and “ψ” is the output image with row (*i*) and column vectors (i,j).

(2) $$\psi (i,j)=C_{i}\left[Max\left(I_{s}^{cs}\right)\right]$$

The third step is illustrated in Equation (3) with a transmission map “T” course and with local minima present inside the image shown in Equation (4).

(3) T(i,j)=1−minj∈λ(i,j)(min×Iscs(z)ACcs)

(4) Av(i,j)=γminj∈λ(i,j)(min×Iscs(z)ACcs)

where $A_{c}$ is the atmospheric light effects, “λ” is the local patch in the color image, “γ” Gamma is constant in Equation (4), zACcs in the dark channel of the image, and “*I*” is the hazy image. “cs ∈ {complimented RGB Image }” Finally, the image is restored using the image restoration model presented in Equation (5).

(5) $$C_{m}(i,j)=\frac{H_{i}(i,j)-\gamma }{\max(T(i,j),t_{i})}+\gamma$$

Further detail of this dehazing approach can be seen from [27]. After dehazing, the image is restored to its original form by using the inverse complement function, as shown in Figure 5b as a dehazed image. The image is then converted into grayscale, shown in Figure 5c, and is ready to process with a virtual hexagonal trellis filter with a single stride convolution function for blood cell image enhancement.

#### 3.1.4. The Proposed Virtual Hexagonal Filter

The proposed method is based on an image enhancement linear kernel, inspired by a square lattice Mean filter. The proposed virtual hexagonal trellis (VHT)-based filter is derived from other filters such as square- and CNN-based filters. The CNN filters are designed over conversion square trellis with nine pixels including full intensity value of neighboring pixels. Neighboring pixels impart a blurring effect over the resultant image. Here, in this approach, we designed a virtual hexagonal trellis over square trellis. The designed approach used seven pixels with a half intensity effect of neighboring pixels. This half intensity of the neighboring pixel reduces the blurring effect from the resultant image. The proposed process is effective in terms of computation cost, which is presented in later section. where the proposed VHT filter’s computational cost with square trellis base filter is compared.

**Figure 6:**
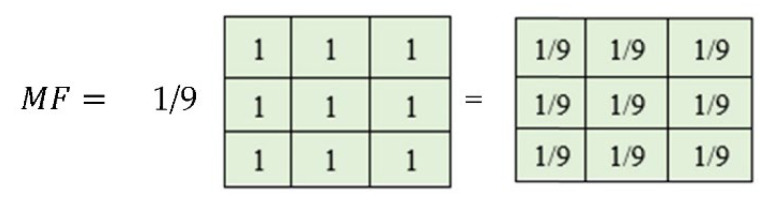
Mean filter 3 × 3 kernel based on a rectangular grid.

The square filter, i.e., Mean filter, is generally used for image enhancement and denoising purposes. A square lattice-based Mean filter is a simple averaging filter used for denoising of images by reducing the high peak intensities of the pixels in an image. Adjusting the high peak intensity in the image improves the brightness of the image at different stages of preprocessing. A 3 × 3 shape-based Mean filter is presented in Figure 6.

**Figure 7:**
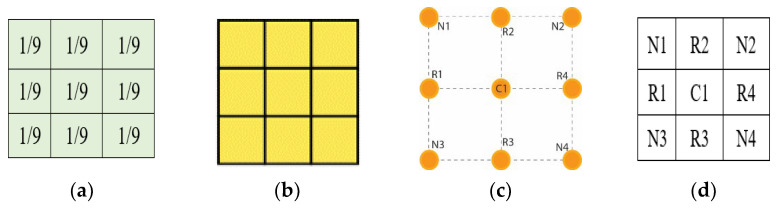
Mean filter square structure (**a**) 3 × 3 averaging filter mask, (**b**) illustration of a square grid in matrix form, (**c**) 3 × 3 square grid structure, (**d**) real and neighboring pixels kernel.

The Mean filter’s intensities are formulated in the form of classical square lattice. The filter here illustrates a 3 × 3 square grid structure. The square grid structure has nine pixels from which four immediate pixels (R1, R2, R3, and R4) are at a unit distance (Figure 7d), whereas the other four neighboring pixels are at a unit plus distance from the central pixel. It is important to mention that, in the literature, these neighboring pixels impart a blurring effect over the central pixel during the enhancement process. Mathematical interpretation is presented to formulate a 3 × 3 filter using nine pixels shown in Figure 7a and its square grid structure in Figure 7b. These nine pixels consist of four neighboring and five real pixels, including the central element in Equation (6) concerning Figure 7c. The proposed filter is inspired by this averaging shape-based 3 × 3 filter in the size presented in Figure 7d.

(6) $$f_{C_{i}}^{\prime }=\frac{1}{9}\left(N_{1}+R_{2}+N_{2}+R_{1}+C_{1}+R_{4}+N_{3}+R_{3}+N_{4}\right)$$

(7) $$f_{C_{i}}^{\prime }=\frac{1}{N}\overset{n}{\underset{i=1}{\sum }}\overset{n}{\underset{j=1}{\sum }}P\left(i,j\right)$$

**Figure 8:**
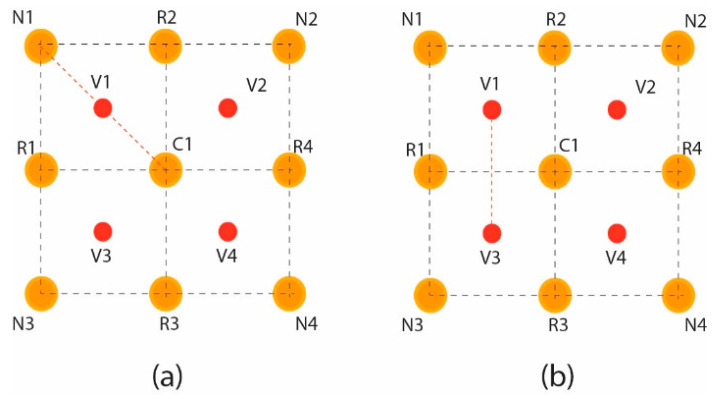
Visualizing virtual pixels of a 3 × 3 filter based on VHT: (**a**) illustration of a square grid in matrix form; (**b**) virtual pixels’ generation.

The pixels’ intensities of the square grid are mapped into the square matrix for the virtual hexagonal concept to formulate virtual hexagonal trellis (VHT). This VHT structure is generated by proposing virtual pixels at half the distance between neighboring and central pixels. The real pixels R1 to R4 from the central pixels are at unity distance, whereas the neighboring pixels are at unity plus distance. Initially, the proposed averaging method is used to formulate a 3 × 3 filter kernel. The proposed method is designed over a sampled 3 × 3 square lattice presented in Figure 8a, and a virtual pixel generation is shown in Figure 8b.

Intensity levels in between the real pixels are not taken for partial effects over the image. The virtual pixels generated at half distances at positions v1, v2, v3, and v4 are calculated using the geometrical average distance $v_{n}=\left(N_{k}+C_{i}\right)/2$ functions. Where *V_n_* is the virtual pixels that are at the center of neighboring pixels *N_k_* and central *C_i_* pixel. From the proposed work, the filter $VHT_{M}F$ is derived from the maximum coverage of hexagonal structure in the circular intensity pixel, and max to min bounds should be defined to fine-tune the filter for maximum effective results.

**Figure 9:**
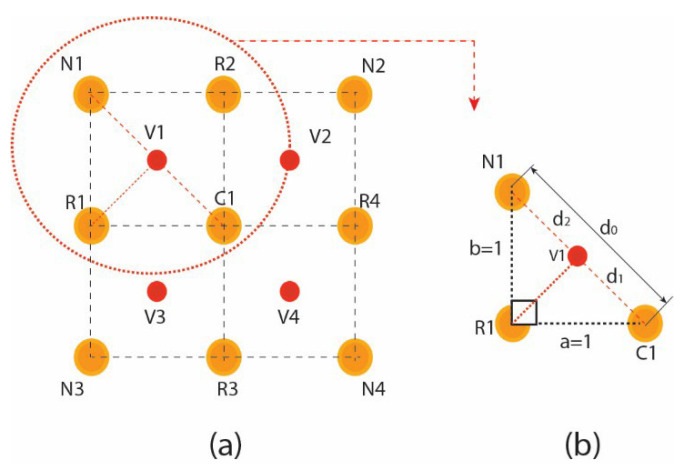
Virtual pixel generation: (**a**) 3 × 3 real pixels square grid; (**b**) average distance calculation.

The Position of v1 shown in the marked circle in Figure 9a is calculated using the average distance of N_1_ and C_1_ in Figure 9b with d_1_ and d_2_, respectively. Intensity to the virtual pixels V_1_ is assigned using the intensity normalization averaging function [29]. This function is often used for brightness adjustment and denoising the subjected images.

**Figure 10:**
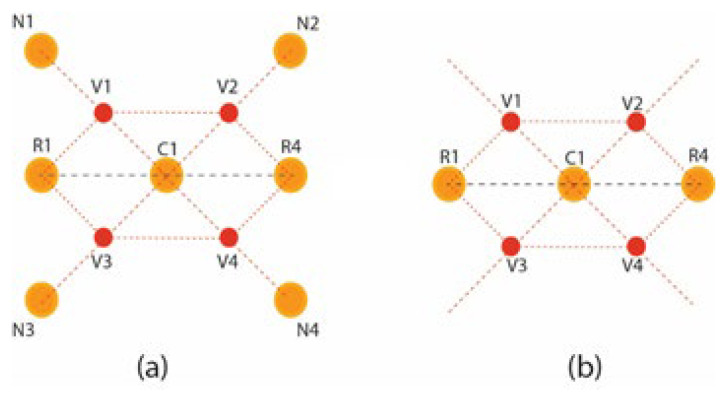
VHT-based structure (**a**) four virtual pixels generated between neighboring and central pixels (**b**) hexagonal lattice for kernel.

**Figure 11:**
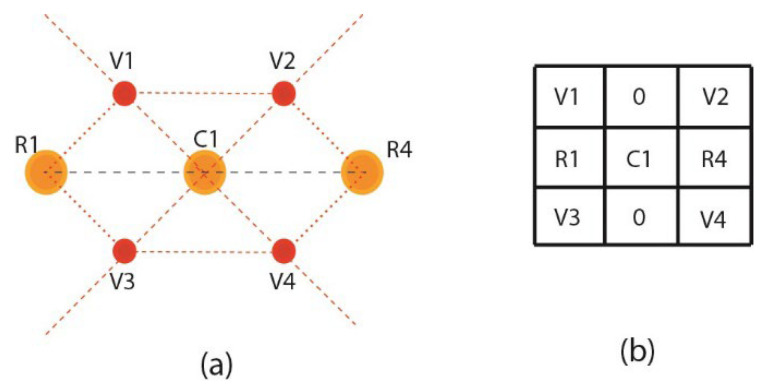
Final seven pixilated structure (**a**) hexagonal trellis structure (**b**) 3 × 3 VHT base kernels.

Intensities to the virtually generated pixels are assigned by averaging the intensities of central real pixels and virtual pixels in Equation (7), as presented in Figure 10a. Central and virtual pixels used for averaging intensities are illustrated over a virtual hexagonal trellis base structure, as presented in Figure 10b. The Hexagonal trellis-based structure in Figure 10b is used to generate a 3 × 3 kernel, as shown in Figure 11b, which convolutes over a dehazed grayscale image for WBC smear image enhancement.

(8) $$f_{C_{i}}^{\prime }=\frac{1}{7}\left(V_{1}+V_{2}+R_{1}+C_{1}+R_{4}+V_{3}+V_{4}\right)$$

Basic variables used to derive VHT-based kernels are presented in Equation (8). *Vi* is the virtual pixels generated at half distance between two pixels that are neighboring and central. “*R*” is the two real pixels at unity distance from the central “*C*” pixel. Cumulative effectiveness of virtual and real pixels is averaged and replaced with the central pixel in a single stride. By simplifying the equation by substituting values of *Vi* in Equation (8), we obtain

(9) $$f_{C_{i}}^{\prime }=\frac{1}{7}\left(\frac{N_{1}+C_{1}}{2}+\frac{N_{2}+C_{1}}{2}+R_{1}+C_{1}+R_{4}+\frac{N_{3}+C_{1}}{2}+\frac{N_{4}+C_{1}}{2}\right)$$

Simplifying Equation (9) by taking Lease Common Multiple (LCM). Average effects of neighboring pixels are imparted over the central pixel in a single stride.

(10) $$f_{C_{i}}^{\prime }=\frac{1}{7}\left(\frac{N_{1}+C_{1}+N_{2}+C_{1}+2R_{1}+2C_{1}+2R_{4}+N_{3}+C_{1}+N_{4}+C_{1}}{2}\right)$$

After LCM, double factor values are assigned to real pixels and central pixels as compared to the other pixels in Equation (10).

(11) $$f_{C_{i}}^{\prime }=\frac{1}{7\times 2}\left(N_{1}+C_{1}+N_{2}+C_{1}+2R_{1}+2C_{1}+2R_{4}+N_{3}+C_{1}+N_{4}+C_{1}\right)\;$$

(12) $$f_{C_{i}}^{\prime }=\frac{1}{14}\left(N_{1}+0R_{2}+N_{2}+2R_{1}+6C_{1}+2R_{4}+N_{3}+0R_{3}+N_{4}\right)$$

**Figure 12:**
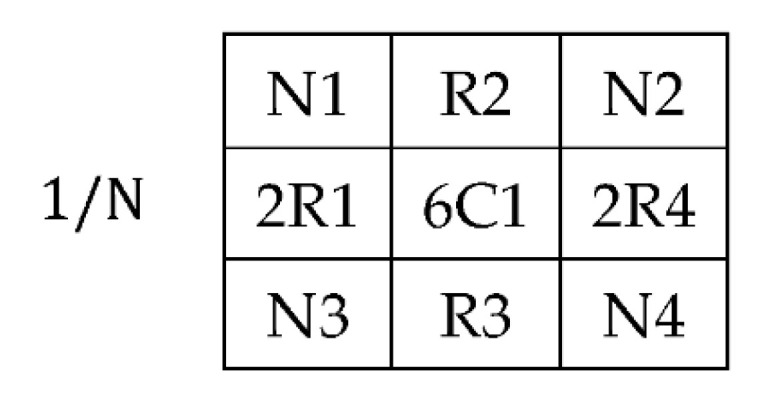
Mask based on 3 × 3 VHT structure.

Image enhancement filters based on virtual hexagonal trellis-based structures ignore the value of real pixels R2 and R3. By ignoring these two real pixels, the computation cost of the proposed method is reduced, and the number of pixels incorporated in the process is also reduced. Therefore, in the proposed kernel value of pixels, R2 and R3 are considered as 0 in the final shape of the VHT 3 × 3 filter. The proposed method is designed on the VHT model inspired by the Mean filter generally used for image enhancement, which reduces the amount of intensity variation between one pixel and the next. It is later used for noise reduction from the image. The filter mask, shown in Figure 12 and designed over the VHT base structure, is convoluted over the dehazed image for enhancement.

(13) $$f_{C_{i}}^{\prime }=\frac{1}{14}\left[\begin{array}{ccc} 1 & 0 & 1\\ 2 & 6 & 2\\ 1 & 0 & 1 \end{array}\right]$$

The convolution method with a single stride length is used from left to right over the complete image. In this process, the image is enhanced using the average function for pixel intensity; moreover, the structure of the filter is refined and converted into a hexagonal lattice. Conceptually, it is an averaging filter shown in Equation (13) for image enhancement; however, it has advantages over square trellis, with fewer pixels. The proposed VHT base filter can be represented in algorithmic form in two steps. First average intensity for a newly generated virtual pixel is calculated from a sample 3 × 3 grid (as represented in Algorithm 1). In the second phase, the derived VHT filter is convolved over input images (Algorithm 2).**Algorithm 1:** Virtual Pixel Generation**1:**  ***Procedure Convolution_VHT_***(***N_i,j_***, ***C_i,j_***, ***R_i,j_ Avg*** )
**2:**   *Initializer* _ *Avg*←0
**3:**  *for each **I_row_** in **I_Input_*****4:**      *for each **I_Col_** in **I_input_*****5:**       *if N_i,j_* == neighbouring pixel and ***C_i+1,j+1_*** == ***central pixel*****6:**              *than Intensity_i,j_* = *add* (*N_i,j_*, *C_i+1,j+1_*)/
**7:**       *Avg* ←*Intensity_i,j_* /*numerOfPixelsUsed*
**8:**       *endif***9:**  *Avg* ←Set intensity to virtual pixel
**10:**       **end procedure**
**Algorithm 2:** Convolution Operation
**1:**  ***Procedure Convolution_VHT_***(***P_i,j_***, ***K_i,j_***, ***E_i,j_ IV*** )
**2:**   *Initializer* _ *variable* ←*IV*
**3:**  *for each **I_row_** in **I_Input_*****4:**     *for each **P_i,j_** in **P_row_*****5:**    *IV* ←*0*
**6:**  *for each **K_row_** in **K_i,j_*****7:**         *for each **K_i,j_** in **K_row_*****8:**       *if **E_i,j_*** ← corresponding Element of Pixel × ***P_i,j_***
**9:**          *than multiply E_i,j_ X **P_i,j_*****10:**       *IV ←add_results***11:**       *endif***12:** IV ←Set output Image pixel
**13:**         **end procedure**

**Figure 13:**
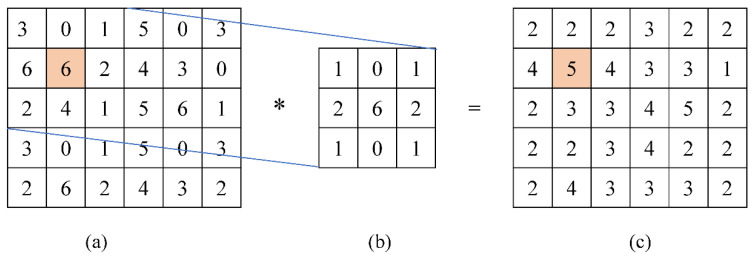
Convolution operation based on VHT base kernel: (**a**) source image pixels, (**b**) VHT base kernel, and (**c**) output image pixels.

In this proposed method, 3 × 3 kernel is used with slight ranges of variables. Averaging effect calculated from the convolution of kernel over the image is assigned to the central pixels after every stride, as shown in Figure 13 and Equation (14).

(14) $$g\left(i,j\right)=\overset{q-1}{\underset{p=0}{\sum }}\overset{p-1}{\underset{q=0}{\sum }}h\left(p,q\right)\times f\left(i-p,j-q\right)\;$$

(15) $$g\left(i,j\right)=\frac{1}{14}\left[\begin{array}{c} \left(3\times 1\right)+\left(0\times 0\right)+\left(1\times 1\right)+\left(6\times 2\right)+\left(6\times 6\right)+\left(2\times 2\right)\\ +\left(2\times 1\right)+\left(4\times 0\right)+\left(1\times 1\right) \end{array}\right]$$

In Equation (14), the VHT kernel in Figure 13b is h(p,q), convoluted over source image pixels’ intensities in Figure 13a, as presented by f(i−p, j−q), and the averaging effect is calculated. The final value after simplification of Equation (15) is 5 as an output pixel intensity g(i,j), as shown in Figure 13c. A similar convolution strategy is adopted to process complete source image pixel values to generate processed output image pixels’ intensities through the VHT kernel.

## 4. Results and Discussion

Image enhancement process outcomes are discussed in this section. Results are acquired using a VHT filter by convolution over the image to impart an averaging effect over each filter. The ALL_IBD2 dataset is used for performance evaluation using different parameters. The presented work is implemented by using a computer system with windows 10 professional, 8 Gb RAM, and 1 TB ROM with 128 MB cache, using MATLAB version 2021(Natick, MA, USA) for a complete evaluation.

### 4.1. Dataset

The publicly available dataset ALL-IBD is used for the parametric evaluation of the proposed method. ALL-IBD contains four classes of WBCs listed in Table 1 as (a) the eosinophils, (b) is the type neutrophils, (c) circular-shaped nucleus type is lymphocytes, and (d) is monocyte. The four classes contain approximately 2400–2500 samples per class with 9954 images in total.

**Figure 14:**
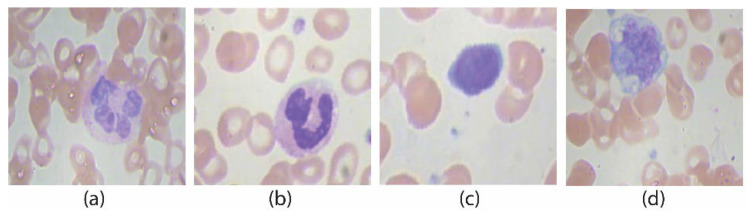
WBCs classes: (**a**) eosinophil, (**b**) neutrophil, (**c**) basophil, (**d**) monocyte.

All images are in RGB format, having images of size 320 × 240. Figure 14 illustrates some sample images of the dataset. The dataset images contain some extra background portions.

### 4.2. Performance Evaluation Measures

The proposed technique is implemented over complete WBC datasets. Mean values of different metrics are measured for performance evaluation. Different performance evaluation protocols are considered to access the proposed method, such as Peak signal noise ratio (*PSNR*) [28,30], the with Mean square error (*MSE*) [28] being measured. The higher the *PSNR*, the better the quality of the image. Similarly, the lower the value of *MSE*, the image has less errors. Mathematically, *MSE* and *PSNR* in are represented in Equations (16) and (17), respectively.

(16) $$MSE=\frac{1}{MN}\overset{M}{\underset{i=0}{\sum }}\overset{N}{\underset{j=0}{\sum }}\left(O\left(i,j\right)-R\left(i,j\right)\right)$$

(17) $$PSNR=10log\frac{255}{\sqrt{MSE}}\pi r^{2}$$

where (*i*,*j*) is the pixel’s position, and “*O*” and “*R*” are original and resultant images. The structure similarity index is another important performance measure. In this parameter, two images are compared in terms of luminance comparison, contrast, and structure comparison, as shown in Equation (18).

(18) $$SSIM_{\left(i,j\right)}=\frac{\left(2\mu_{x}\cdot \mu_{y}+C_{1})(2\sigma_{xy}+C_{2}\right)}{\left(\mu_{x}^{2}+\mu_{y}^{2}+C_{1})(\sigma_{x}^{2}+\sigma_{y}^{2}+C_{2}\right)}$$

where $“\mu_{x}”$ is the average of “x”, $“\mu_{y}”$ is the average of “y”, $“\sigma_{x}^{2}”$ is the variance of “x”, $“\sigma_{y}^{2}”$ is the variance of “y”, and “C” is the stabilizer for the weak denominator. Equation (19) presents the normalization cross coefficient (*NCC*), which estimates the difference between the common interest point.

(19) $$NCC=\sum_{i=1}^{m}\sum_{i=1}^{n}\frac{\left(O_{ij}\times R_{ij}\right)}{O_{ij}^{2}}$$

where “*O*” and “*R*” are the two comparative original and resultant images. Normalized absolute error (*NAE*) reflected in Equation (20) is also used to access our approach. The higher value of the *NAE* shows the poor quality of the image.

(20) $$NAE=\frac{\sum_{i=1}^{m}\sum_{i=1}^{n}\left(\left|O_{ij}-R_{ij}\right|\right)}{\sum_{i=1}^{m}\sum_{i=1}^{n}\left(O_{ij}\right)}$$

Computational cost (CC) analyzes that, if the cost is higher, the method is inefficient compared to the method which has a lower computational cost. Some parameters related to the image enhancement are also evaluated using the VHT filter over the WBC dataset.

(21) $$EME=\underset{\varphi \;\epsilon \left\{\varphi \right\}}{\max}x\left(\frac{1}{k_{1}k_{2}}\overset{k_{2}}{\underset{m=1}{\sum }}\overset{k_{1}}{\underset{n=1}{\sum }}20\;log\frac{I_{max;k,l}^{\mu }}{I_{min;k,l}^{\mu }}\right)\;$$

where $I_{min;k,l}^{\mu }$ and $I_{max;k,l}^{\mu }$ are the minimum and maximum of the image inside a unique block $w_{k,l}(i,j$). {φ} is the class of orthogonal transforms used for image enhancement after splitting it into $k_{1}k_{2}$ blocks. These spatial domain performance evaluation measures include a measure of the enhancement by entropy (*EME*) [31], mean absolute error (*MAE*) [32], blind/reference less image spatial quality evaluator (BIRSQUE) [33,34], naturalness image quality evaluator (NIQE) [35], and blind image quality index (BIQI) [35,36]. The proposed work showed the advantage over classical square domain algorithms in terms of low computational complexity. Reduced storage is used during square to hexagonal transformation, and, finally, it uses only seven pixels to process, whereas, in convention denoising methods, nine pixels are used for processing every stride of the convolution of the filter.

### 4.3. Experiment 1: VHT Filter Analysis Based on Constant Factor Variation

**Figure 15:**
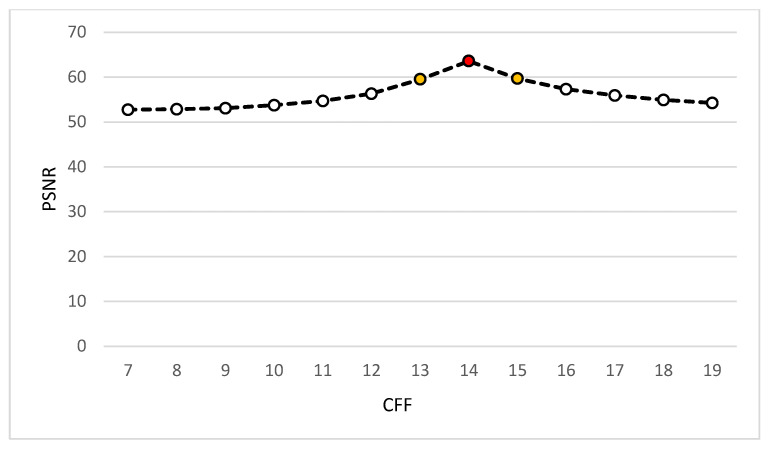
PSNR concerning CFF.

**Figure 16:**
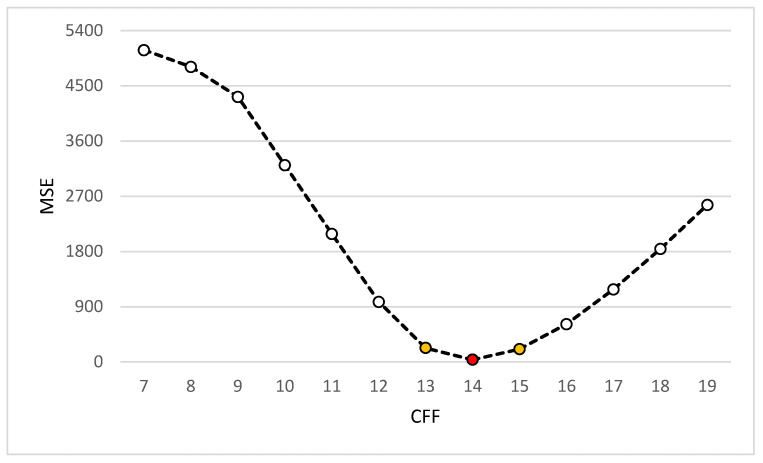
MSE concerning CFF.

The proposed VHT base filter shows some improved results when the constant factor of the filter (CFF) kernel is vacillated from values 7 to 19 as the multiplication factor of the kernel in Table 2. At CFF 7, the intensity converges towards the bright region, and no internal regions of WBCs are seen. Whereas, when the CFF value increases from 7 to 19, it shows the cytoplasm and other objects inside it clearly, as shown in Figure 15. It is observed that, at CFF 14, clear regions are seen, and the best qualifying values are recorded, as shown in Figure 15 and Figure 16.

During the analysis of the VHT filter, PSNR values at different ranges are calculated. The PSNR value at FCF 14 shows the best ratio value shown in red circle and the second-best value is presented in yellow circle in Figure 15, which shows the best quality of image at this point.

Similarly, the VHT filter shows the lowest value of MSE as compared to the other points of FCF in Graph 16. A lower value of MSE at FCF value 14 reflects the very low error ratio and high-quality image at that point presented in red circle and the second best value is shown in yellow circle in Figure 16.

### 4.4. Experiment Based on VHT

**Figure 17:**
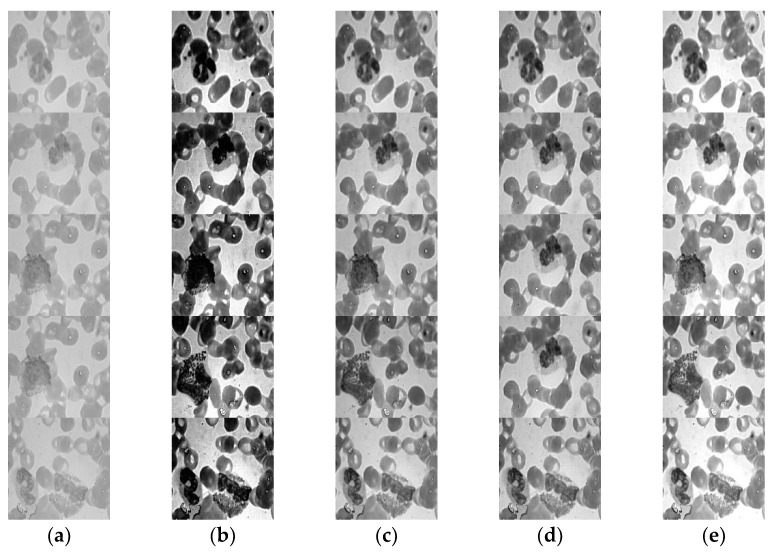
Image samples visualizations after employing various enhancement techniques: (**a**) original gray image, (**b**) histogram equalization, (**c**) Mean filter, (**d**) Winner filter, (**e**) proposed VHT filter.

In this section, visualization effects after experimenting with over-generalized images of WBC are shown for medical image enhancement. The proposed method shows improved results shown in Figure 17e of medical images, which shows information preservation during the implementation of the proposed method. Here, the proposed approach is specifically designed for WBC image enhancement. These images can further undergo the infection detection process. Therefore, it is necessary to enhance these WBC images for better image analysis in the future. The proposed method shows significantly improved visual results, as shown in Figure 17, in comparison with previous methods. It is to be noted that numeric data extracted after implementation of the proposed method are rounded up to the nearest value to aid in the readability and understanding of results. In Figure 17a, a simple input image at the grayscale level is shown. Figure 17b reflects the visual results when the histogram equalization technique is implemented over the WBC image. Similarly, Figure 17c–e shows the enhancement results of Mean, Winner, and VHT filters, respectively. From all results, the proposed VHT base enhancement filter showed the best results in terms of PSNR, MSE, and SSIM.

The proposed virtual hexagonal structure is compared with classical Mean and Winner filters, and improved results over the standard dataset were found. After a comparative analysis was performed, the PSNR of the proposed method was 60.687, and HE was evaluated up to 58.11; Winner filter was 53.03 and the Mean filter PSNR over ALL-IBD dataset was 58.18. The VHT-based filtering method in Table 3 shows the best results in terms of PSNR.

**Figure 18:**
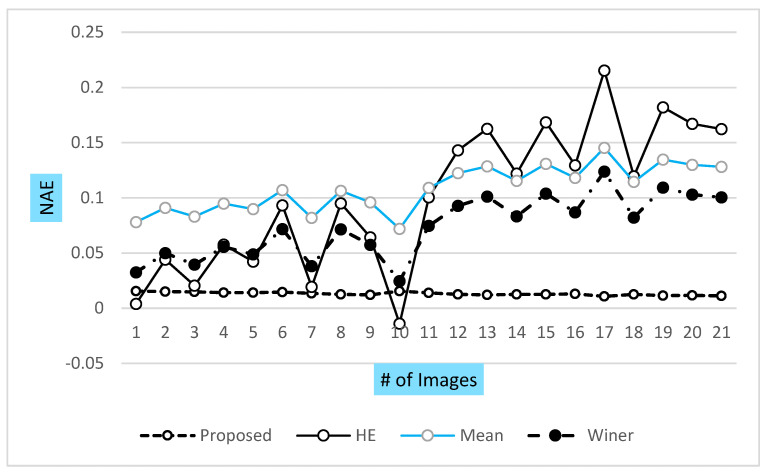
NAE of proposed and other classical techniques.

Similarly, the proposed method VHT-based filter shows MSE 947.20, SSIM 0.924, and entropy 7.90 as the best results when using the ALL-IBD dataset over existing filtering-based methods. Furthermore, the VHT filter shows PSNR values of 30.782, 29.779, and 60.687 when applied over a standard dataset and radiological and WBC datasets, respectively. The PSNR value shows the high quality of the image compared to the existing methods. Similarly, MSE values of 84.542, 29.122, and 947.20 present a low signal error ratio when implemented over standard, radiological, and ALL-IBD datasets, respectively. On the other hand, the VHT filter found in the 2nd to 3rd position when SSIM values were 0.8, 0.739, and 0.924 are collected over presented datasets in Table 3, and the best values of SSIM are collected from the Mean filter, which is 1.00, which dominated over the proposed method. Finally, entropy values were 7.016 and 6.529, and the best value of VHT was 7.016 over standard, radiological, and WBC datasets (ALL-IBD). The overall results of the VHT filter over WBC image enhancement showed improved results. PSNR results, in comparison with other classical methods, are presented in Figure 18. A higher value of PSNR presents a high-quality image.

**Figure 19:**
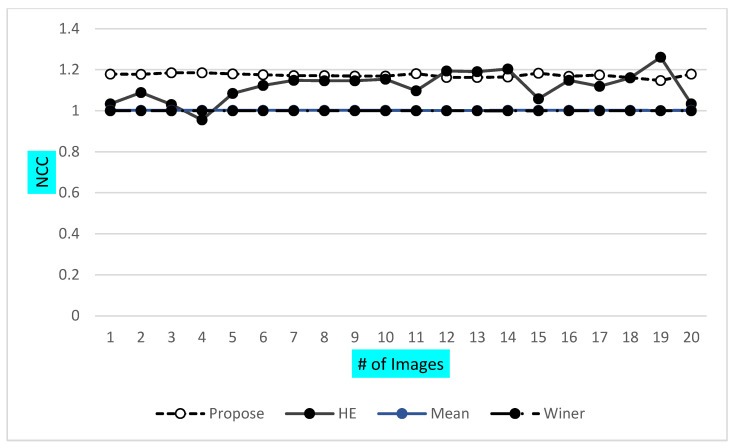
NCC of proposed and other classical techniques.

The presented graph in Figure 18 shows that the VHT filter provided the best NAE over the other classical filter during WBC image enhancement. Similarly, the high value of the normalized cross coefficient reflects the high quality of the image when compared with other classical methods. VHT has a high value over the Mean and Winner filter. In this paper, normalized absolute error and computational time are also discussed for an in-depth analysis of the proposed method. The presented VHT filter showed very low NAE and computational time. Similarly, some other parameters also tested against the reference and test images of the ALL-IBD dataset for the refined evaluation of the proposed method, which includes the normalization cross coefficient (NCC) shown in Figure 19, which shows a similar measure, used to estimate the difference of the interest points of the resultant and reference images.

**Figure 20:**
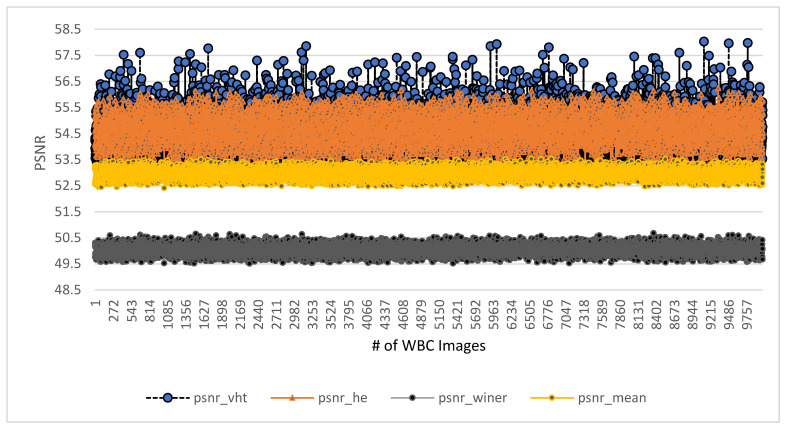
Comparison of proposed and other image enhancement methods in terms of PSNR.

The final observation is listed as the final evaluation in terms of computational time. All other square structure-based methods are also tested along with the proposed VHT structure-based filter for image enhancement. A brief visualization in terms of the PSNR of proposed and other previous methods is shown in Figure 20. Total images of the ALL-IBD dataset are 9999, including all classes.

**Figure 21:**
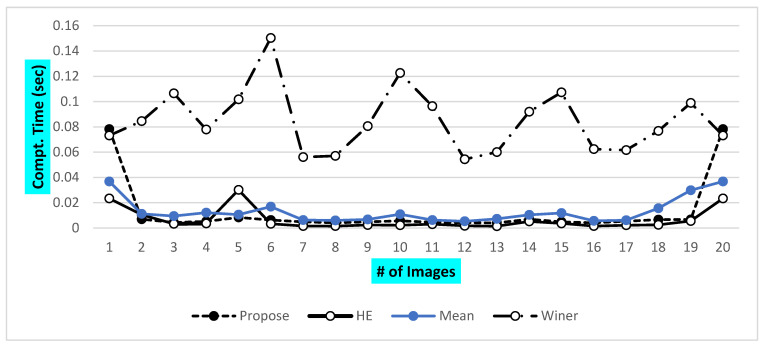
The computational cost of proposed and other classical techniques.

The range of the PSNR of all these images for the proposed method varies from 55.5 to 58.3, and the PSNR of classical methods is below the minimum range of the VHT filter-based proposed method. The Winner filter, Mean filter, and histogram equalization method showed PSNR from 49.5 to 51.5, 52 to 53.5, and 53 to 55.9 respectively. This visualization presents a higher PSNR value of our proposed method in comparison with previous methods for image enhancement. Finally, a comprehensive analysis of all efficient methods is presented in Table 4. The different parameter is considered for comparison as per availability from the literature (Figure 21).

**Figure 22:**
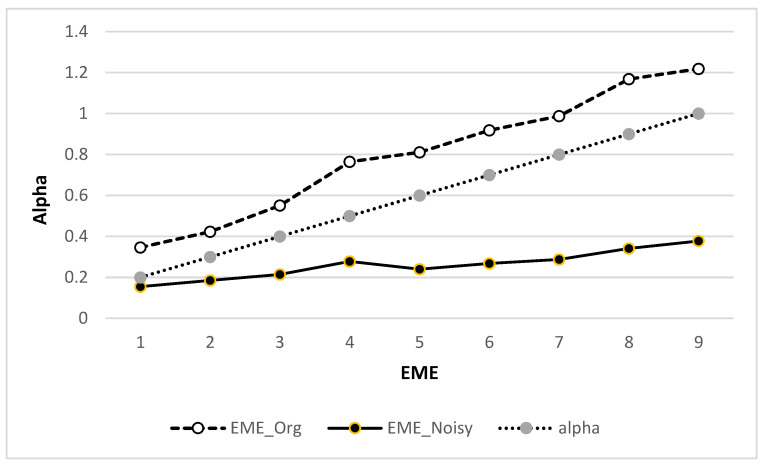
Measure of enhancement (EME) vs. alpha value.

Values of alpha in Figure 22 vary from 0.1 to 1 to value the effect of the VHT filter for enhancement. The value of alpha increases the EME of the WBC original image compared to the noisy image.

The proposed virtual hexagonal trellis-based filter dominates over the classical method based on a square grid structure. The VHT filter shows the best PSNR of 63.580 and, on seconds level, the MT-Kapur method exits. The SSIM value of the proposed method is approximately near the original structure stability value, which is 1.

The comparison of alpha values over the noisy and original WBC image and other enhancement parameters are listed in Table 5 and Table 6, respectively.

Some enhancement parameters are also measured to refine the analysis VHT filter for WBC images’ enhancement.

The parameters listed in Table 5 are analyzed during WBC image enhancement using constant factor “alpha” in the alpha rooting algorithm. Similarly, some of the spatial domain parameters for image quality assessment are listed in Table 6 for comparative analysis.

## 5. Discussion

Proposed method implementation leads to outcomes. These outcomes are extracted in the form of results. Evaluation parameters used for image enhancement analysis are Peak Signal to Noise Ratio (PSNR), MSE (Mean square error), SNR (signal to noise ratio), Structural Similarity Index (SSIM), and entropy. It gauges the spread of the intensity range among the pixels. A higher value of the entropy of an image compared to any other image signifies greater distribution of intensity values among all the pixels in an image. Factors and numerical range values related to standard MSE, PSNR, and entropy are discussed in the literature. All the above enhancement measurement factors are well known in the literature, and only entropy is used for the extra verification and validation of image enhancement. Entropy is the disorder of any system, and image processing is used to check how many extra values are added to the image for image enhancement.

These extra values may be considered as increasing resolution factors in an image. These said qualitative and quantitative parameters are calculated over different datasets. The virtual hexagonal structure over square trellis is obtained and satisfies two main important properties of the real hexagonal structure of images. Firstly, the properties for every neighboring pixel for the central pixel, and, secondly, consistent connectivity with the neighboring pixel to pixel relationship/mathematical ratio. Consistency and connectivity are calculated using a conventional trigonometric identities function for distance from central to neighboring pixels. In this work, the individual comparison of qualifying parameters is also discussed in Table 3, where PSNR and MSE show the efficient outcomes of the proposed method. The proposed method is implemented over our focused dataset ALL-IBD WBC images. Publicly, an available dataset is of a low resolution and a blurry nature. Direct use of this dataset with any proposed method will result in inaccurate results. Proposed VHF schemes show dramatically improved results in the case of enhancement and region contrast adjustment, as well as a visual and statistical enhancement of the results of the ALL-IBD dataset. These obtained results, averaged with the whole dataset’s combined effect, can be illustrated. We have calculated a minimum to maximum enhancement concerning the original image in terms of percentage. During the processing of the image, the results summarized in Figure 20 presented that the proposed method is not only visually effective in enhancing the image, but also processes the image ten times fast as compared to the classical methods. Initially, when the proposed that the VHT structure-based filter is convoluted over the target image, as it took a time of 0.08 ms; however, when the sequence started, it processed all images with an average processing time of 0.0045 msec, which shows that the presented method is too cost-effective in terms of computational cost, as shown in Figure 21.

In Table 4, all classical methods are presented with qualifying parameters in comparison with the proposed method. All presented methods are implemented over WBC images for enhancement. Commonly used qualifying parameters are cited in different papers, i.e., PSNR, MSE, SSIM, and entropy and spatial domain performance evaluation parameters (EME, MAE, BRISQUE, NIQE, and BIQI) of all images for quality assessment are discussed in Table 3, Table 5 and Table 6. An overview of these methods is listed, which clearly shows the outperformance of our proposed VHT method. PSNR is very high compared to other methods over the same dataset. Similarly, SSIM is also at a high peak which shows the structural uniformity in the VHT method.

## 6. Conclusions

In this paper, an image enhancement filter over a square grid using the concept of the hexagonal trellis is proposed. The proposed work also dominates over the existing HE, Mean, and Winner filter approaches used for image enhancement. We employed a VHT base filter instead of a square lattice base filter because it solved the blurring and contrast problems and improved the enhancement results. We validated our proposed method based on the ALL-IBD WBC dataset with histogram equalization, Mean filter, and Winner denoising filters. The proposed VHT-based filter condenses storage issues, computation load, and information lost while converting the square lattice into the hexagonal trellis. The proposed method based on the virtual hexagonal approach contributes to all kinds of images generally and specifically—it is very suited for the enhancement of medical (WBC/Radiology and pathologically) images for diagnostic purposes. The experimental results presented that the proposed enhancement filter outperforms the state-of-the-art enhancement methods and generates a more realistic image in terms of patterns and texture details. The presented approach is efficient with less computational complexity and storage needs simultaneously. There is a minor transformation cost (information loss) compared to the classical approach in square trellis, which will be handled in the future.

## Figures and Tables

**Table 1:** ALL-IBD WHITE BLOOD DATASET (Type: RGB, Size: 320 × 240).

Classes	Number of Images
Eosinophils	2497
Neutrophile	2483
Lymphocytes	2478
Monocyte	2499

**Table 2:** PSNR and MSE in terms of (CFF).

Org. Image	CFF	Filter Variation	PSNR	MSE	Output Image
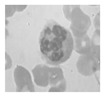	7	$f_{c_{i}}^{\prime }=\frac{1}{7}\left[\begin{array}{ccc} 1 & 0 & 1\\ 2 & 6 & 2\\ 1 & 0 & 1 \end{array}\right]$	52.76	5084	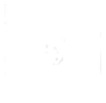
	8	$f_{c_{i}}^{\prime }=\frac{1}{8}\left[\begin{array}{ccc} 1 & 0 & 1\\ 2 & 6 & 2\\ 1 & 0 & 1 \end{array}\right]$	52.88	4810	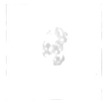
	9	$f_{c_{i}}^{\prime }=\frac{1}{9}\left[\begin{array}{ccc} 1 & 0 & 1\\ 2 & 6 & 2\\ 1 & 0 & 1 \end{array}\right]$	53.12	4322	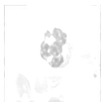
	10	$f_{c_{i}}^{\prime }=\frac{1}{10}\left[\begin{array}{ccc} 1 & 0 & 1\\ 2 & 6 & 2\\ 1 & 0 & 1 \end{array}\right]$	53.76	3210	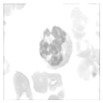
	11	$f_{c_{i}}^{\prime }=\frac{1}{11}\left[\begin{array}{ccc} 1 & 0 & 1\\ 2 & 6 & 2\\ 1 & 0 & 1 \end{array}\right]$	54.69	2090	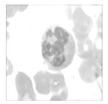
	12	$f_{c_{i}}^{\prime }=\frac{1}{12}\left[\begin{array}{ccc} 1 & 0 & 1\\ 2 & 6 & 2\\ 1 & 0 & 1 \end{array}\right]$	56.34	978.6	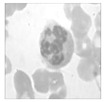
	13	$f_{c_{i}}^{\prime }=\frac{1}{13}\left[\begin{array}{ccc} 1 & 0 & 1\\ 2 & 6 & 2\\ 1 & 0 & 1 \end{array}\right]$	59.51	226.3	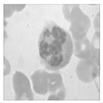
	**14**	$f_{c_{i}}^{\prime }=\frac{1}{14}\left[\begin{array}{ccc} 1 & 0 & 1\\ 2 & 6 & 2\\ 1 & 0 & 1 \end{array}\right]$	**63.58**	**34.81**	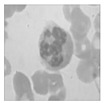
	15	$f_{c_{i}}^{\prime }=\frac{1}{15}\left[\begin{array}{ccc} 1 & 0 & 1\\ 2 & 6 & 2\\ 1 & 0 & 1 \end{array}\right]$	59.69	209.4	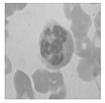
	16	$f_{c_{i}}^{\prime }=\frac{1}{16}\left[\begin{array}{ccc} 1 & 0 & 1\\ 2 & 6 & 2\\ 1 & 0 & 1 \end{array}\right]$	57.34	618.8	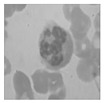
	17	$f_{c_{i}}^{\prime }=\frac{1}{17}\left[\begin{array}{ccc} 1 & 0 & 1\\ 2 & 6 & 2\\ 1 & 0 & 1 \end{array}\right]$	55.93	1185	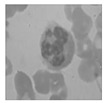
	18	$f_{c_{i}}^{\prime }=\frac{1}{18}\left[\begin{array}{ccc} 1 & 0 & 1\\ 2 & 6 & 2\\ 1 & 0 & 1 \end{array}\right]$	55.93	1842	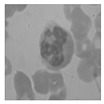
	19	$f_{c_{i}}^{\prime }=\frac{1}{19}\left[\begin{array}{ccc} 1 & 0 & 1\\ 2 & 6 & 2\\ 1 & 0 & 1 \end{array}\right]$	54.96	2584	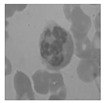

**Table 3:** Parametric analysis of VHT Filter.

Matric	Dataset	Proposed	HE	Winner	Mean
**PSNR**	Standard [37]	**30.782**	30.119	29.893	27.728
	Radiology [38]	**29.779**	29.038	28.835	26.795
	ALL-IBD [39]	**60.687**	58.11	53.03	58.18
**MSE**	Standard	**84.542**	95.377	102.250	168.03
	Radiology	**29.122**	31.907	36.423	61.549
	ALL-IBD	**947.20**	3465	32.443	3019
**SSIM**	Standard	**0.800**	0.767	0.774	0.638
	Radiology	**0.739**	0.730	0.706	0.580
	ALL-IBD	0.924	0.679	0.651	**1.000**
**Entropy**	Standard	7.016	**7.091**	6.993	6.996
	Radiology	6.529	**6.539**	6.531	6.501
	ALL-IBD	**7.90**	6.59	5.55	6.20

**Table 4:** Comparison with existing works on ALL-IBD dataset.

Reference	Methodology	Dataset	PSNR	MSE	SSIM
[40]	Stochastic fractal	ALL-IBD	32.390	0.6991	0.97
[41]	SFS		35.010	0.6874	0.93
	PSO		33.500	-	0.92
	K-Mean		34.023	-	0.89
	ABC		34.040	-	0.93
[42]	Mean Shift		28.600	0.6654540	0.85
[43]	MT-Kapur		57.810	0.3566527	-
[44]	Leb-TLBO		56.550	-	-
**Proposed**	**VHT filter**		**63.580**	0.5583	0.985

**Table 5:** Comparison of alpha vs. EME.

Alpha Value	Alpha Rooting [31]	EME Org. Image	EME Noisy Image
0.2	-	0.346114	0.154574
0.3	-	0.422835	0.184626
0.4	-	0.550855	0.214108
0.5	-	0.764463	0.277344
0.6	-	0.811176	0.23959
0.7	156.249	0.9186	0.268161
0.8	2.35060	0.987115	0.287299
0.9	-	1.168741	0.340932
1.0	-	1.21844	0.377405
1.1	-	1.40244	0.459378

**Table 6:** Results of parametric analysis.

Img	EME	MAE	BIRSQUE	NIQE	BIQI
1	0.3461	6.42535	38.7352	6.0575	0.204
2	0.3161	6.32578	38.8486	5.6807	0.18944
3	0.3310	6.30827	41.6925	5.2208	0.18721
4	0.4121	6.35861	42.458	5.5765	0.22813
5	0.3446	6.41299	37.3479	5.2374	0.23094
6	0.3275	6.45363	39.3929	6.0202	0.19619
7	0.2843	6.39611	37.8443	5.3732	0.17665
8	0.3065	6.42818	39.0292	5.5498	0.18398
9	0.2662	6.42976	39.6043	5.4301	0.17439
10	0.2807	6.36987	37.9429	6.0695	0.17802

## Data Availability

Exclude this statement.
